# Stop smoking advice by practice assistants after routine cervical screening in general practice: A qualitative exploration of potential barriers and enablers

**DOI:** 10.1080/13814788.2022.2053105

**Published:** 2022-04-08

**Authors:** Marthe B. L. Mansour, Matty R. Crone, Henk C. van Weert, Niels H. Chavannes, Kristel M. van Asselt

**Affiliations:** a Department of General Practice, Amsterdam UMC, Academic Medical Centre Amsterdam, Amsterdam, The Netherlands; b Amsterdam Public Health, The Netherlands; c Department of Public Health and Primary Care (PHEG), Leiden University Medical Centre, Leiden, The Netherlands

**Keywords:** General practice/family medicine, general, qualitative designs and methods, prevention, addiction and abuse

## Abstract

**Background:**

Cervical screening could be an appropriate routine moment to provide female smokers with tailored stop smoking advice. In Dutch general practice, cervical smears are performed by practice assistants.

**Objectives:**

This study was performed in preparation for a randomised trial to identify potential barriers and enablers for a brief stop smoking strategy performed by trained practice assistants after routine cervical screening.

**Methods:**

Between December 2016 and March 2017 three focus group meetings were held with ten practice assistants, three nurses, and six general practitioners to explore their views and expectations towards the proposed approach. We analysed data using thematic analysis. Identified factors are presented within the framework of the Social-Ecological Model.

**Results:**

Potential barriers and enablers were identified at individual, interpersonal, and workplace levels. Practice assistants, nurses and GPs did not consider assistants to have a role in stop smoking care. They believed it is feasible to register smoking status but had reservations towards providing advice by assistants, for which knowledge and skills are needed. Practice assistants’ own beliefs about smokers and smokers’ response to stop smoking advice might influence how assistants and smokers interact. An explanation of why advice is given could help, provided assistants have enough time and experience with the smear. The nurses’ availability and general practitioners’ view on prevention might affect the delivery of the strategy by the assistant.

**Conclusion:**

At individual, interpersonal, and workplace levels, several factors could influence the provision of a stop smoking strategy by a practice assistant.

KEY MESSAGES

- Practice Assistants (PAs) conduct preventive tasks like cervical screening but do not deliver stop smoking advice.
- PAs, nurses and general practitioners feel reservations to stop smoking advice by PAs after routine cervical screening.
- The nurse’s availability and general practitioner’s view on prevention could influence the provision of the strategy.

## Introduction

Opportunities to give stop smoking advice are underused in general practice [1], even though guidelines recommend that all smokers should be given relevant advice and especially those smokers at high risk of, or with complaints related to, tobacco-related disease [2]. Smokers without signs or symptoms of smoking are less likely to be given stop smoking advice [1,3]. This is a missed opportunity because the preventive impact of smoking cessation on tobacco-related disease and early death increases if smokers quit at a younger age [4]. Cervical cancer screening could be an opportunity to routinely provide female smokers with stop smoking advice and support, as the profits of quitting at this relatively young age are big [5,6]. In females, 12% of new cancer cases are attributable to smoking [7]. Smoking is a risk factor for continued high-risk human papillomavirus (hrHPV) infection of the cervix [8]. In addition, evidence suggests that tobacco and nicotine promote oncogenic mechanisms in cervical cells [9].

In the Netherlands, women aged 30–60 years are invited for cervical screening at their general practice every 5 years. In this age category, approximately 1 in 5 to 6 of women are daily smokers [10]. In 2019 around 413.000 Dutch women had their smear taken.

In the Netherlands, the national cervical cancer screening programme is carried out in general practice and the smear is performed by a practice assistant (PA) while stop smoking advice or support is mainly delivered by general practitioners (GPs) and/or by qualified nurses. Although studies on the provision of stop smoking care (SSC) by auxiliary healthcare workers (such as practice assistants (PAs)) are sparse, there is evidence that advice given by the assistant is as effective as interventions by doctors and nurses [11–13]. As PAs are employed in most Dutch general practices, they could have a potential role in registering smoking status, giving stop smoking advice, or referring patients to cessation counselling.

There is, however, no evidence on the effectiveness of this approach in women who undergo a routine cervical smear. In a cluster randomised trial, we plan to investigate the effect of a stop-smoking strategy delivered by PAs after cervical screening in general practice.

When designing and implementing complex interventions, such as health behaviour interventions, it is important to identify barriers and enablers among the patients and the healthcare professionals involved. For this purpose, qualitative research methods are the most suitable [14]. Previously, we conducted an interview study among female smokers to explore the prospective (i.e. anticipated) acceptability of the approach [15]. In this study, we identified potential factors that could act as barriers or enablers to the delivery of stop smoking advice by a PA after routine cervical screening, among involved healthcare professionals. The results will be used to optimise the design of the trial and process evaluation.

## Methods

### Study design and participants

This qualitative study was conducted prior to the onset of a cluster randomised trial that studies the effect of a stop-smoking strategy delivered by practice assistants (PAs) after cervical screening in general practice. Focus group meetings were held to identify potential barriers and enablers to the delivery of the strategy to optimise trial design and identify elements for the process evaluation. The focus group approach was chosen to obtain views from different perspectives and stimulate discussion among involved staff, who have different roles in general practice: PAs, nurses and general practitioners (GPs).

In Dutch general practice the general practitioner (GP) is responsible for providing stop smoking advice or support, and is the practice assistant (PA)’s and nurse’s employer. Nurses are regularly involved in disease management programmes and deliver stop smoking care (SSC). They can act as the expert of SSC in a practice. Our intervention, however, will be delivered by PAs, who perform cervical smears but are currently not engaged in delivering stop smoking advice. In daily practice, PA’s and nurse’s role may overlap, see Box 1 explaining their role in Dutch general practice.

The trial strategy is based on the Ask-Advise-Connect method [16]. The PA provides brief stop smoking advice consisting of: 1. Ask about smoking status, 2. Provide brief stop smoking advice, and 3. Actively offer an appointment for support given by the nurse or GP. Two main aspects of the strategy could differ from care as usual: i) the advice is given by a PA, ii) the advice is given after a cervical smear.

We invited PAs, nurses and GPs for these focus groups. We aimed to have heterogeneous groups but predominantly include PAs in the focus group meetings, as the intervention primarily centres around their activities. Nurses and GPs were also invited as they are the PA’s direct colleagues and employer and because they have a role in SSC in general practice. *Via* their contribution to the focus group meetings, we intended to gain a more realistic view on potential interaction of nurses and GPs with the PA in the future trial (as opposed to focus group meetings with only PAs). Purposive sampling ensured a variation in age, work experience, and type of general practice. Also, four participating assistants had recruited female smokers to participate in a separate qualitative interview study [15] and for this reason had practiced in asking women about their smoking status after a smear (not in trial setting). Participants were invited *via* a recruiting message on social media and recruiting talks after training programmes. No PAs, nurses and/or GPs from the same practice simultaneously participated in a focus group. All participants were Dutch and currently employed in a Dutch general practice. Written consent was obtained. Participants received a gift voucher (€25) as compensation for their time.

Box 1 Dutch general practice: practice assistants and nurses

**Table:** 

The core team of a Dutch general practice consists of a general practitioner (GP), a practice assistant (PA), and – in most practices - a qualified nurse. In countries with similar healthcare systems, PAs are known as ‘medical assistants’, ‘medical secretaries’, ‘healthcare assistants’ or ‘allied health personnel’. The professional education for PAs consists of a three-year vocational training (‘Middelbare Beroepsopleiding’ (MBO) level four, equivalent of European Qualifications Framework (EQF) level four), which is comparable to the ‘BTEC Level three Extended Diploma’ in the U.K. or VET-diploma in Denmark. Activities for PAs vary according to practice needs. In the job description published by the Dutch National Association of General Practitioners (Landelijke Huisartsen Vereniging, *LHV*), an assistant’s tasks broadly cover the intake or triage of patients and providing assistance for medical or administrative and organisational tasks. Many PAs have their own consultations, for example: taking cervical smears or measuring blood pressure [*i*]. The knowledge, skills, and competence of PAs vary in EU member states [*ii*], which can partially explain differences in the deployment of PAs between countries. An average Dutch general practice employs a 1.94 full-time equivalent PA and a 0.63 fte nurse [*i*]. Job qualifications for practice assistant differ from those of a qualified nurse (professional education for nurses is required, at EQF level six). Qualified nurses provide protocol-based care and are specialised in chronic care management, typically for patients with diabetes, COPD / asthma, cardiovascular diseases, or vulnerable elderly [*iii*]. For example, the nurse can address issues such as medication use, but also engages in lifestyle counselling such as smoking cessation. Nurses work more or less independently. In general terms, nurses provide care for specific patient groups in general practice and typically do not engage in administrative tasks. In contrast, in some PAs also provide chronic care management or other case-based tasks which can overlap with those of a qualified nurse.

*i* Landelijke Huisartsen Vereniging, https://www.lhv.nl/service/functiewaardering-huisartsenzorg, Handleiding FWHZ_Functiewaardering Huisartsenzorg.pdf, 2019.

*ii* Kroezen M, Schafer W, Sermeus W, *et al.* Healthcare assistants in EU Member States: An overview. Health Policy. 2018 Oct;122(10):1109-1117.

*iii* Nederlandse Zorgautoriteit, https://puc.overheid.nl/doc/PUC_3629_22, Praktijkkostenonderzoek huisartsen 2015.pdf, 2017.

### Data collection

Three focus group meetings were held between December 2016 and March 2017. A focus group guide was developed to stimulate discussion and explore the following topics: 1) Organisation, tasks, and experiences of participants with SSC in general practice, 2) Attitudes towards stop smoking advice given by a PA after cervical screening, and 3) Expected barriers to, and enablers of, this approach. By means of these topics, we attempted to address the future acceptability and feasibility of the strategy’s content and its provision in daily practice. At the start of each focus group meeting, the outline of the strategy (Ask-Advise-Connect) was explained (Table 1). Focus groups were moderated by two female researchers ((KvA (GP and PhD) and MC (PhD)) with experience in qualitative methods and research on smoking cessation. One female researcher (MM (MD and PhD-student)) with experience in qualitative methods acted as an observer and did not participate in the group discussions. The meetings lasted 70–90 min. Data were collected until no new themes emerged from the focus group sessions.

**Table 1.** Guide with topics used in the focus group discussions with practice assistants, nurses and general practitioners ^a^.

*1. Opening of the focus group* - Short introduction: what is your profession, where do you work? - Provide a brief answer to the following question: ‘How is smoking status registered within your practice?’
*2. Explanation of the strategy, explanation of the Ask-Advise-Connect steps* ‘We would like to know how you think about the delivery of a stop smoking strategy by the practice assistant after routine cervical cancer screening in general practice. This strategy consists of three steps: asking and registering smoking status, providing brief stop smoking advice, referring smokers to the practice nurse for smoking cessation counselling.’
*3. First response to the stop smoking strategy* - What are your first thoughts on this strategy? - What would be a reason (not to) choose this approach? - How do you think your colleagues think about this approach?
*4. In depth discussion* *Health behaviour* - What are the tasks of the practice assistant / practice nurse / general practitioner when it comes to health behaviour change? *Smoking cessation* - Who’s tasks is it to register smoking status / provide stop smoking advice / deliver counselling? - How do you work with colleagues when you provide stop smoking support? - What are your experiences with smokers and with stop smoking in patients? *Smoking cessation after cervical screening* - How would you feel about delivering stop smoking advice after the cervical smear? - How do you expect female smokers would respond to such advice? - What do you believe could be the problems and/or benefits of this approach?

^a^summarised and translated from original version in Dutch.

### Analysis

Focus group meetings were audio-recorded, transcribed verbatim, reviewed for accuracy, and imported into MAXQDA 12. Thematic analysis was used, going from open to analytical coding and with an iterative process of data collection and analysis [17]. After each focus group session field notes and topics were discussed. Between sessions the transcripts with open codes were discussed. This resulted in minor adjustments to the topic list, for example an elaboration on the acceptability of the strategy. KvA and MM coded the first focus group meeting independently; MM coded the other two. Three researchers (KvA, MC, MM) read all transcripts and discussed codes, categories, and themes in detail. Discrepancies were discussed until consensus was reached.

The exploration adopted a focus on the PA, from the perspective of the different healthcare professionals. During the coding and the analysis we explicitly looked at who provided the information (PA, nurse or GP). First, we focussed on the PA’s perspective and subsequently on the perspective of nurses and GPs. This was done for the following reasons: little is known about the role of PAs in SSC, in contrast to nurses and GPs; the strategy centres around activities of the PA in which the GP and nurse are not always engaged (such as performing smears). Finally, the identified factors were categorised based on their influence on stop smoking care by the PA; for this purpose, we used a social-ecological framework [18]. The framework enabled us to group the identified factors at the individual, interpersonal and organisational levels. Also, within the different levels, we determined which of the steps of the stop smoking strategy (Ask, Advise, Connect) these factors could impact. We believe this structuring will enable us to translate the results to learning points for trial design and identify point of focus for the process evaluation. We report our study using the 32-item checklist of consolidated criteria for reporting qualitative research (COREQ) [19].

## Results

### Participant characteristics

In total 10 practice assistants (PAs), 3 nurses, and 6 general practitioners (GPs) participated (Focus group 1: 3 PAs, 2 nurses, 2 GPs; Focus group 2: 2 PAs, 3 GPs; Focus group 3: 5 PAs, 1 nurse, 1 (general practitioner (GP)). Participant characteristics are listed in Table 2.

**Table 2.** Participant characteristics.

Characteristics	Participants
	(*n* = 19)
Gender	
Female	17/19
Male ^a^	2/19
Age	27–60 yrs.
Profession	
Practice assistant	10/19
Nurse	3/19
General practitioner	6/19
Geographical location of general practice	
Major city	11/19
Urbanised region	6/19
Rural/village	2/19
Smoking status ^b^	
Ex-smoker	10/19
Never-smoker	9/19
Years of working experience ^c^	
1	2/19
2–10	4/19
> 10	13/19
Attended courses	
Stop smoking support	5/19 ^d^
Motivational interviewing	5/19 ^e^

^a^Two male participants were both GPs, all other participants were female.

^b^Three categories: Current-smoker, Ex-smoker and Never-smoker.

^c^Total number of working years as a practice assistant/nurse/general practitioner.

^d^No practice assistants previously participated in a course on providing stop smoking support.

^e^One practice assistant previously participated in a course on motivational interviewing techniques.

### Factors of influence

Factors are presented at the level of influence: the individual level (namely, the practice assistant (PA)), the interpersonal level (interaction between assistant and female smoker), organisational level (work environment, e.g. the general practice). For each level, we indicate at which step of the stop smoking strategy the factors are applicable: Ask, Advise and/or Connect. The illustrative quotations are listed in Table 3.

**Table 3.** Illustrative quotations.

Level of influence & potential factors		Illustrative quotations (*PA ^a^, GP ^b^*)
Individual level – the practice assistant	The practice assistant’s professional role	***#19-PA:*** *At our practice a person’s smoking status is not registered, at least it can’t be seen immediately. Actually, we have little to do with it. Well, apart from when they say that they want to stop smoking and ask questions about how to go about it. Then we refer the patient to the practice nurse.* ***#21-PA:*** *First you complete the form for cervical screening: ‘Do you have symptoms. Do you smoke?’ It could easily be integrated.* *** #17-Nurse:*** *I was thinking that it would generate resentment to ask about smoking after the smear. But the practice assistants from my practice said they would ask for it right away, if needed.* *** #21-PA:*** *Because you can easily explain it, because of the relationship with HPV.* ***#1-Nurse:*** *My idea would be to put the question for smoking status on the form (for cervical screening). And to provide smokers with a leaflet or something. I believe it is quite a burden for practice assistants to have to provide this whole stop smoking advice.* ***#4-PA:*** *Yes, it was quite a challenge to go about it […] Those who smoke want to know why you ask. We would need interview techniques, to learn how to structure the consultation.* ***#22-PA:*** *Motivational interviewing, that is a must. You know, that you get trained in how you approach people.* ***#6-PA:*** *Yes, that you understand why people smoke and don’t start to get negative. You should be able to formulate it nicely.* ***#9-GP:*** *You have to think through how to approach female smokers. Simply asking for the smoking status should not at all be a problem for practice assistants […] But for the advice, maybe a chart with information could help. Also to inform the patients.* ***#16-PA:*** *I would prefer to only work with the questionnaire, “do you smoke, yes or no”? And that the nurse takes care of the rest, the advice and counselling and all that.* ***#11-GP:*** *Asking the question should not be the problem. But getting an extensive training. I don’t know. I think they (Pas) are too busy.*
	Attitude towards the cervical smear as a teaching moment	***#5-PA:*** *I believe we need to make women conscious of the risks, using that specific moment […] The percentage of female smokers is high. It can serve as a moment of realisation to make it personal.* ***#9-GP:*** *I would be glad if the practice assistant took her time to record the smoking status as a way to make patients aware of the risks. I believe many people are aware of lung cancer and cardiovascular disease. Honestly, the risk of cervical cancer in smokers wasn’t so evident to me either.* ***#18-GP:*** *Many young women do not get in touch with our practice because they are healthy or only for their children. So once they are here [for the smear] you make use of that moment.*
Interpersonal level – interaction between practice assistant and female smoker	Do not overwhelm	***#16-PA:*** *First, they are in a vulnerable position, literally. The explanation that they probably need at that moment: What does it (smoking status) have to do with it (smear test)? And their anxiety and stress at that moment.* ***#3-Nurse:*** *I think you really take them by surprise. As I see it, I believe it is better to ask permission to discuss smoking. You could even do this before the cervical smear.*
	Challenging switch from smear to smoking	***#4-PA:*** *I can imagine that it can be quite a challenge if you have only recently started performing cervical smears. And having to switch from the smear to asking about that (smoking), you’re not going to be able to do it in merely 10 min.* ***#1-Nurse:*** *If there is a link, you can obviously use it. Women come for the cervical smear but they smoke. These things are at odds with one another, which you can easily explain.* ***#11-GP:*** *I believe it is good to take your time to explain the relationship between smoking and cervical cancer. And to provide some background information. In case difficult questions are asked, you can provide an answer to that.*
	Beliefs about smokers and their response to stop smoking advice	***Various PAs:*** *The enhanced risk of cancer in smokers could stimulate them (smokers) to quit, and: Women could be receptive to quit advice because many of them have or want to have children or: Having an aberrant smear result would make women more receptive to quit advice, versus: Women who visit for the smear and have not experienced any major health-related problems will not change their behaviour.* ***#8-GP:*** *If they smoke they want to hear that they don’t have cancer. On the other hand, I don’t think they want to stop (smoking) to avoid it. They just want to hear that they don’t have it – they can then go on smoking. Stopping is a stage further, but this is true for all addictions.* ***#10-GP:*** *I recognise having thoughts such as: ‘it’s useless to discuss smoking because it is not the reason for their visit.’ I am actually surprised how often smokers are open to it, or at least thinking about it or dealing with it.*
Workplace – organisation and attitudes at the general practice level	Nurse’s interest and availability	***#20-PA:*** *It’s noticeable that people like it if they immediately get an appointment. ‘An appointment, that’s OK.’ Because our practice nurse is only here on Mondays and Tuesdays. I think she doesn’t like to give stop smoking advice.* ***#18-GP:*** *The same in our practice; she didn’t choose to do it. I believe the nurse is the only bottleneck.* ***#17-Nurse:*** *You could schedule an appointment directly after the smear, right?* ***#25-PA:*** *Yes, but our nurse needs to confirm this. And if she is not motivated to do it. I mean, she should be willing to cooperate in this, for easy referral.*
	General practitioner’s view on prevention	***#21-PA:*** *I talked to the boss about it once. He said: ‘You can lower cholesterol levels by 40% if you stop smoking.’ And then I said: ‘Well, then you’ve got something to do.’ Then he said: ‘But people have to choose to do so’ I was shocked about this, that you could influence cholesterol levels by 40%. I think that he was setting out the strategy. I was looking for – what do we think about this? As a practice?*
	Within-practice approach to stop smoking care	***#7-GP:*** *I don’t consider us fit for primary prevention, or at least, it’s not a task for us. […], I will not start talking about smoking behaviour with a patient with knee problems. The same goes for weight, I won’t start to discuss that if someone comes with a cough. Sometimes, I try to address it if the subject is related.* ***#10-GP:*** *As a doctor, I see the importance of primary prevention in this relatively young group of people. I see that as a positive aspect of being a GP. That you can pick out certain patients and send them to the practice nurse and advice: do you smoke – yes or no? If you smoke, do you want to do something about it?*

^a^PA: practice assistant.

^b^GP: general practitioner.

### Individual level – the practice assistant

Factors apply to ‘Ask’ and ‘Advise’.

#### The PA’s professional role

PAs did not consider themselves to have a professional role in stop smoking care (SSC), neither did the nurses and GPs, considering that such advice was part of the nurses’ core business. A few assistants registered smoking status occasionally, such as with new patients or during blood pressure control. None of the assistants routinely provided stop smoking advice and support. Nurses and GPs confirmed this also was the case in their practices.

Regarding the *ask-advise-connect* steps, PAs thought that asking about and registering a woman’s smoking status would not be a problem: it was considered a simple extra question that could easily be integrated. Some nurses and GPs expected that the PA would not want to ask for the smoking status. However, all GPs and nurses believed PAs would be capable of assessing the smoking status, with some GPs considering this to be a simple way to register smoking status in women who do not often visit the practice.

PAs thought that giving actual stop smoking advice would be challenging as it requires additional time, skills, and knowledge. GPs and nurses were also not sure whether assistants would be capable of providing this advice, as this is not something they routinely engage in and could be time-consuming. Furthermore, PAs and GPs believed training would be needed to learn how to provide advice. On the other hand, assistants thought they generally had more time and were more approachable for patients than GPs and nurses.

Some of the assistants were willing to be more involved in SSC but did not know how. Several assistants sometimes talked briefly about lifestyle advice during their consultations, such as talking about stress, diet or smoking behaviour or referred a smoker to the nurse. None of the assistants knew the actual content of SSC.

#### Attitude towards the cervical smear as a teaching moment

Most participant PAs, nurses and GPs were not aware of the correlation of smoking and the development of cervical abnormalities. Nearly all focus group participants agreed that in order to use the smear as a legitimate teaching moment, a clear link or proven association between smoking and the cervical smear or cervical cancer should exist. PAs and several GPs considered it important to make women aware of the health risks of smoking.

### Interpersonal level – interaction between practice assistant and female smoker

Factors apply to ‘Advise’ and ‘Connect’.

#### Do not overwhelm

PAs, nurses and GPs thought it necessary to explain why women are asked about their smoking habit just after a smear test. Most PAs noticed that women were tense before a smear and would not want them to become more stressed.

The PAs also anticipated that women would ask why they were being asked about smoking at that moment and, therefore, PAs wanted to have an adequate answer.

#### Challenging switch from smear to smoking

All participants believed addressing health behaviour is difficult and time-consuming. In general, PAs, nurses and GPs perceived it as easier to discuss smoking cessation if women could be told of the connection between smoking and cervical cancer.

The four PAs who had briefly talked about smoking after a smear test said that smokers reacted positively. However, these assistants considered it challenging to switch from taking a smear to talking about smoking cessation. Having enough time and experience performing smears were deemed essential to feeling confident about performing these activities.

#### Beliefs about smokers and their response to stop smoking advice

Smoking was considered an addiction but from their comments, we deduced that some participant PAs and GPs would not necessarily approach smokers as such. For example, some PAs said that smoking is the smoker’s responsibility and that failing to quit is a rational choice. Conversely, participating nurses showed more knowledge on how to address smoking behaviour.

The participants had different expectations about the acceptability and effectiveness of the stop smoking strategy. PAs related this to the health status of female smokers, for example, expecting a positive response to advice due to increased risk perception. One GP reported being surprised by the fact how often smokers are open to discussing the subject of smoking, in contrast to what healthcare providers expect. Other PAs expected less effect on stopping smoking as this was not the reason for the visit. Some GPs argued that accepting a conversation is not a guarantee for action. The absence of health-related problems might imply an absence of motivation to stop smoking leading to high no-show rates for smoking cessation counselling. For the same reason, two nurses expected low referral numbers after the smear.

### Workplace level – organisation and attitudes at the general practice level

Factors apply to ‘Connect’ and to the delivery of the strategy as a whole.

#### Nurse’s interest and availability

The nurse was considered a potential bottleneck as her availability to provide smoking cessation counselling might limit referral. Also, the nurse’s motivation to guide smokers in smoking cessation was expected to influence the quality of support delivered. All participating nurses were motivated for SSC, but some PAs and GPs experienced that the nurse in their practice had special interest in other aspects of chronic disease management than smoking behaviour.

#### General practitioner’s view on prevention

PAs expected the GP to lead the way regarding SSC and prevention strategies. Some assistants argued that prevention is part of primary care; others wanted to be aware of health behaviour risks to be able to inform patients about their health risks. However, despite their own views, PAs would follow the course set by the GP.

#### Within-practice approach to stop smoking care

Some participants reported that their practice adopted a ‘reactive’ approach to smokers – the patient should ask for advice and support. General practitioners in these practices did not consider primary prevention (such as offering advice or support in case of smoke addiction in patients without smoking-related disease) and health promotion activities to be their task, for example, viewing it a task imposed by the government.

Other practices had a more ‘proactive’ policy. For example, all newly registered smokers were offered stop smoking support and quitters were actively monitored. GPs in these practices considered primary prevention (such as treatment of smoke addiction in patients without smoking-related disease) and health promotion activities to be a task for general practice.

## Discussion

### Main findings

In this focus group study we found that, at the individual level, practice assistants (PAs), nurses and general practitioners (GPs) did not consider PAs to have a professional role in stop smoking care (SSC). They believed asking for the smoking status to be feasible but had more reservations towards providing advice by PAs, for which training in knowledge and skills would be needed. To use the smear as a legitimate teaching moment a clear link between smoking and the cervical smear should exist.

When it comes to the interaction between practice assistant (PA) and patient, PAs expect the switch from smear to smoking to be challenging. PAs, nurses and GPs stated that enough time and experience with performing smears are needed, and a clear explanation to patients why smoking is discussed. Also, PAs’ own beliefs towards smokers and smokers’ response to stop smoking advice might influence the interaction between smoker and PA. Across all professions, some participants believed an absence of health-related problems or the reason for the visit not being stopping smoking might imply an absence of motivation of female smokers, which could influence how smokers respond to the strategy. Nonetheless, PAs valued having advice at hand to inform patients about health risks.

Nurse’s interest and availability could influence referral for counselling. PAs tend to follow the course on SSC and addiction to smoking set by the general practitioner (GP), with either positive or barrier attitudes towards smoking cessation existing at the workplace level.

### Comparison with prior work

#### PA’s role and task in stop smoking care

PAs did not consider themselves to have a professional role in SSC. PAs, GPs and nurses were not sure whether PAs would be capable of providing advice. One observational study and two studies using data from RCTs showed that stop smoking advice or counselling given by assistants is at least as effective on smoking cessation outcomes or patient satisfaction as similar care provided by registered nurses or GPs [11–13]. Also, in contrast to our findings two qualitative studies conducted in the U.K. reported that GPs and nurses consider smoking cessation a suitable task for PAs [20, 21]. A possible explanation for these findings [11–13] could be a difference in range of tasks for PAs between the UK and the Netherlands. Although GPs and nurses were previously interviewed on PA involvement in smoking cessation [20, 21], no prior qualitative explorations are available that included PAs and report on their professional role, experience, expectations or needs towards SSC in general practice.

PAs valued the smear as a potential moment to inform patients about health risks. To provide advice, PAs, nurses and GPs thought more time, skills and knowledge were required. This need is supported by the results of two reviews and one RCT that assessed the effects of, and factors of influence on, the implementation of health promotion interventions. The authors stress the need for training and change in practice culture to effectively engage PAs in primary prevention [22–24].

### Interaction between PA and female smoker

A recent cross-sectional survey study assessed willingness to receive lifestyle advice during cancer screening. Of cervical screening participants (*n* = 768), 78.9% were willing to receive advice [25]. Participants in our study were unsure whether the cervical smear is a legitimate teaching moment [8]. The smear differs from other teaching moments, such as a cardiovascular disease consult, considered appropriate because there is a clear relationship with smoking [26] and it is a less sensitive moment, which can be the case during cervical smear visits. The PAs, nurses and GPs believed an explanation on why smoking is discussed is needed, as it was expected patients would request this and it could facilitate the switch from smear to ‘smoking’. Also, PAs considered the stress of the smear. There is overlap between the PAs’ views and those of female smokers who were previously interviewed to assess the prospective acceptability of the approach. Female smokers requested an explanation of why smoking behaviour is addressed. Other factors of influence on smokers’ acceptability were feeling at ease during the smear test and a non-judgemental approach when discussing smoking cessation [15]. This latter request from female smokers underlines the need to address attitudes towards smokers and expectations of the approach when training PAs to deliver a stop-smoking strategy.

### Colleagues and practice culture

As for practice culture, participants described either a reactive or proactive approach towards smokers within their practice. Our findings that barrier attitudes towards smoking cessation still exist in general practice overlap with previous findings that SSC is not always considered a core task in primary care [1, 27]. Prevention has been identified as one of the five core tasks of Dutch GP’s [28]. This includes ‘indicated prevention,’ which among others aims to identify individuals with the risk factor of smoking addiction without related disease [28]. However, so-called ‘universal prevention’, for example, the prevention of taking up smoking, has not been identified as a task for Dutch GPs.

We found that a stop smoking strategy needs support from all healthcare professionals involved. PAs will refer smokers to the nurse and PAs tend to follow the course set by the GP. Looking at similar findings from this field, it is very likely that the GP and nurse especially impact the PA’s delivery of the stop smoking strategy in terms of support, priority, and availability of time or resources [23, 24, 29].

### Strengths & limitations

In this qualitative study, mainly PAs participated, which was the result of our purposive sampling strategy to explore their views and expectations. The number of focus group participants may be limited because three different types of professionals participated. Yet, we believe the number of conducted focus groups was adequate to prospectively obtain a broad exploration of relevant views and expectations towards the proposed approach that centres around the PA. As we predominantly invited PAs and focussed on their perspective in the analysis, it could be that viewpoints from GPs or nurses have been underexposed. Furthermore, the participation of GPs and nurses enabled an interactive approach with the identification of factors of influence at different levels (individual, interpersonal, workplace). In the presence of nurses and GPs it could be that PAs talked less openly. To minimise this risk, we did not invite PAs with GPs or nurses from the same practice to participate in a session. Also, the moderators actively stimulated all participants to contribute to the discussion. The study participants might have had a positive attitude towards SSC, whereas more sceptical professionals may not have responded to the request for study participation.

### Implications

The results provide new information on how involved healthcare professionals view the PA’s professional role and needs in SSC, on their views and expectations to combining a stopping smoking strategy with the cervical smear, as well as the influence of all disciplines involved on the delivery of the strategy.

The findings will be used for the trial as follows; on the *individual and interpersonal level:* 1. PAs should participate in a training that enhances their knowledge and skills on how to provide brief advice; 2. The training should address PAs’ beliefs towards smokers and how smokers might respond to advice; 3. Example sentences should be developed for PAs to facilitate the advice and explain to women why smoking is addressed after the smear; on the *workplace level:* 4. Nurses should be stimulated to participate in the training to support PAs and plan on how smokers will be referred for counselling within the practice.

For the process evaluation of our future trial focus areas should be as follows; on the *individual level:* 1. Perceived role and actual tasks of PAs in SSC, PA’s skills and knowledge, attitudes and expectations towards the strategy; on the *interpersonal level*: 2. Available time, experience performing cervical smears, beliefs about smokers and how they will respond to the strategy, and an evaluation of the actual experiences during the trial when delivering the strategy with the switch from smear to smoking (*such as: what did and what did not work?; how did women respond?),* on the *workplace level*: 3. Availability, cooperation with and involvement of the nurse and GP, the practice culture and course set by the GP on smoking cessation, support of direct colleagues, prioritisation and availability of time and resources [23, 24, 29].

The future experiences from PAs, nurses and GPs (and female smokers) in trial setting will provide qualitative information on barriers and enablers for further implementation and compare the strategy with care as usual. Previous literature shows the potential of PAs’ deployment in SSC [11–13]. Considering the number of (younger) female smokers that might routinely be reached when visiting their general practice for the cervical smear, the PA’s deployment for this specific opportunity deserves further attention.

## Conclusion

This focus group study with PAs, nurses and GPs identified several factors at individual, interpersonal and workplace levels that could influence the provision of a stop smoking strategy by a PA after routine cervical screening in general practice. The results can be used to develop a stop smoking intervention delivered by the PA or a similar intervention combined with cervical cancer screening.

## Supplementary Material

Supplemental Material
